# Smart Glass Film Reduced Ascorbic Acid in Red and Orange Capsicum Fruit Cultivars without Impacting Shelf Life

**DOI:** 10.3390/plants11070985

**Published:** 2022-04-04

**Authors:** Xin He, Sachin G. Chavan, Ziad Hamoui, Chelsea Maier, Oula Ghannoum, Zhong-Hua Chen, David T. Tissue, Christopher I. Cazzonelli

**Affiliations:** 1 National Vegetable Protected Cropping Centre, Hawkesbury Institute for the Environment, Western Sydney University, Locked Bag 1797, Penrith, NSW 2751, Australia; xin.he@westernsydney.edu.au (X.H.); s.chavan@westernsydney.edu.au (S.G.C.); c.maier@westernsydney.edu.au (C.M.); o.ghannoum@westernsydney.edu.au (O.G.); z.chen@westernsydney.edu.au (Z.-H.C.); d.tissue@westernsydney.edu.au (D.T.T.); 2 School of Science, Western Sydney University, Penrith, NSW 2751, Australia; 20215428@student.westernsydney.edu.au; 3 Global Centre for Land Based Innovation, Western Sydney University, Hawkesbury Campus, Richmond, NSW 2753, Australia

**Keywords:** Smart Glass Film, glasshouse, shelf life, postharvest, ascorbic acid, cuticle, capsicum, carotenoid, crop nutrition, environment, energy efficient

## Abstract

Smart Glass Film (SGF) is a glasshouse covering material designed to permit 80% transmission of photosynthetically active light and block heat-generating solar energy. SGF can reduce crop water and nutrient consumption and improve glasshouse energy use efficiency yet can reduce crop yield. The effect of SGF on the postharvest shelf life of fruits remains unknown. Two capsicum varieties, Red (Gina) and Orange (O06614), were cultivated within a glasshouse covered in SGF to assess fruit quality and shelf life during the winter season. SGF reduced cuticle thickness in the Red cultivar (5%) and decreased ascorbic acid in both cultivars (9–14%) without altering the overall morphology of the mature fruits. The ratio of total soluble solids (TSSs) to titratable acidity (TA) was significantly higher in Red (29%) and Orange (89%) cultivars grown under SGF. The Red fruits had a thicker cuticle that reduced water loss and extended shelf life when compared to the Orange fruits, yet neither water loss nor firmness were impacted by SGF. Reducing the storage temperature to 2 °C and increasing relative humidity to 90% extended the shelf life in both cultivars without evidence of chilling injury. In summary, SGF had minimal impact on fruit development and postharvest traits and did not compromise the shelf life of mature fruits. SGF provides a promising technology to block heat-generating solar radiation energy without affecting fruit ripening and marketable quality of capsicum fruits grown during the winter season.

## 1. Introduction

Rising populations and crop nutrient deficiency are substantial worldwide issues that require sustainable solutions [1,2,3]. Protected cropping facilities provide precise environmental control to promote year-round high yielding crop production [4], yet energy consumption can be a limiting factor [5]. Recent advances in innovative covering material technologies can reduce glasshouse energy consumption while transmitting sufficient light to maintain crop production [6,7]. While most studies evaluating covering materials for greenhouse crop production have focused on reporting crop growth and yield, few studies have investigated the impact of cover materials on quality. For example, covering materials tested on greenhouse production of tomato [8], cucumber [9] and lettuce [10] displayed variable effects on fruit development and quality formation among cultivars depending upon the cover and seasonality. Thus, the impact of altered light by covers on fruit quality needs to be assessed based upon seasonal variation.

Smart Glass Film (SGF) or Ultra-Low-Reflectivity-80 (ULR-80) is a commercial tinting product that has a low thermal emissivity coating designed to permit most of the useful light and block heat-generating radiations. It was shown that a capsicum crop growing within a high-tech glasshouse in a temperate Australian climate containing HD1AR diffuse glass consumed 70% of energy for heating compared to 30% for cooling, with heating being predominant in spring and autumn [11]. During both cool and warm climate conditions, SGF coated onto HD1AR diffuse glass helped to reduce cooling energy and fertigation demand of an eggplant crop grown in a glasshouse yet did not significantly affect ventilation or heating energy use [12]. SGF blocked 85% of ultraviolet, 58% of far-red, and 26% of red light, causing a 19% reduction in photosynthetically active radiation (PAR) and 25% reduction in eggplant fruit yield [13]. The reduction in yield could be attributed to reduced photosynthesis and a lower xanthophyll de-epoxidation state (DPS) which affected nonphotochemical quenching and the optimum response to oscillating changes in the light environment [13,14]. During the cultivation of Red and Orange capsicum fruit cultivars under SGF a reduction in ultraviolet (UV) light (69%; 221–279 nm), red light (26%; 600–699 nm), far-red light (51%; 710–850 nm), and PAR (19%; 400–700 nm) correlated with lower yields [15]

Altering the light quantity and spectral quality can have profound effects on fruit development, ripening, and yield, as well as micronutrient levels [16,17,18,19,20]. For example, plants grown under flexible photovoltaic rooftop panels with 10% shading showed a reduction in tomato size [21]. Similarly, a dye-sensitized solar cell covering material technology that reduced illuminance by 20% delayed tomato fruit ripening [22]. Supplementation of far-red light can enhance growth rate during early stages of tomato development [23], while a reduction in far-red transmittance affects sugar and organic acid content [17].

The accumulation of antioxidants such as carotenoid pigments can be affected by changes in light diffusion, UV radiation, and the quality of the light spectrum [24,25]. It was previously shown that SGF impacted carotenoid composition and the DPS of the xanthophyll cycle in eggplant leaves [13]. The eggplant fruit mesocarp appears whitish-green and accumulates only trace levels of carotenoids [26], making it difficult to assess the impact of SGF on fruit pigment levels [13]. Capsicum (*Capsicum annuum* L.) is a self-pollinating crop with diverse genetic variance in fruit colours of green, yellow, orange, and red that depends upon which carotenoid accumulates as the end product in mature fruit [27,28,29]. Therefore, capsicum fruit varieties provide a valuable resource to assess the impact of SGF on fruit antioxidant levels and pigmentation.

Postharvest shelf life can be extended by reducing water loss in fruit crops through the modification of the postharvest storage environment. The epidermal cuticle layer is the primary barrier to water loss, especially in capsicum fruit that contain fewer stomata [30,31,32]. Decreased UV light and shading negatively impact hydrophobic cuticle formation, leading to water loss and a shorter shelf life [32,33]. Water loss accelerates the loss of nutritional value and causes fruit softening, leading to excessive shriveling [34]. Fruit firmness correlates to fruit weight, pericarp thickness, and epidermal cuticle thickness [31,35]. Decreasing temperature and increasing relative humidity are commonly used to control water loss and maintain postharvest fruit quality, whilst trying to avoid chilling injury [36,37]. The impact of SGF on fruit water loss under different storage requirements and hence shelf life remains unclear.

We report the impact of SGF on fruit development, and postharvest quality traits, such as ascorbic acid, total soluble solids, fruit colour, and carotenoid accumulation in Red and Orange capsicum cultivars grown during the winter season. The cuticle thickness, water loss, and firmness of fruits stored under different temperatures and relative humidity (RH) were assessed to ascertain potential effects that the SGF could have on fruit shelf life. This study broadens knowledge of the potential impacts of SGF on marketable attributes of capsicum fruits, thereby benefiting protected cropping industries seeking to improve energy use sustainability.

## 2. Results

### 2.1. SGF Impacted Red Fruit Cuticular Deposition but Not Development at Mature Ripe Stage

**Figure 1:**
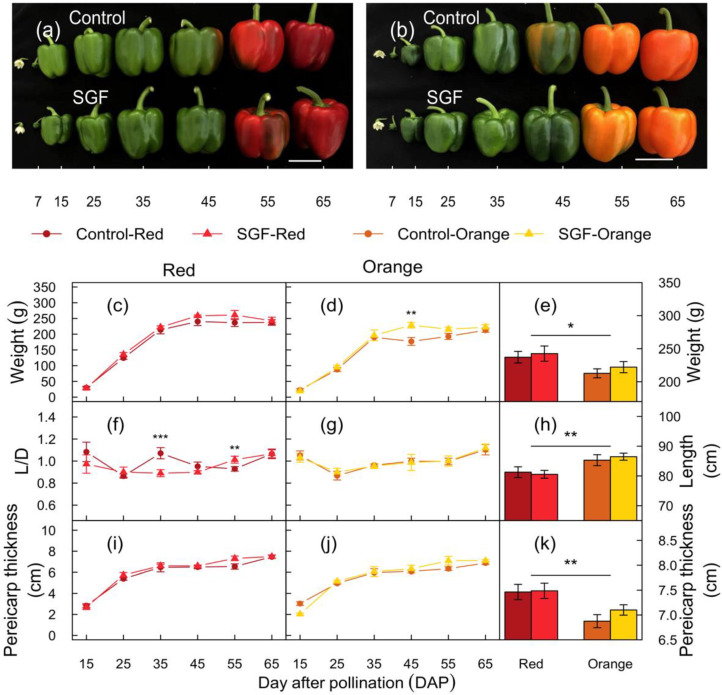
Morphology and development of fruits from plants grown under the SGF. (**a**,**b**) Photos showing morphological features of representative Red (**a**) and Orange (**b**) cultivar fruits harvested from plants grown under SGF or diffuse control glass. Images depict different stages of fruit development (7 to 65 DAP). Scale bars (5 cm) are displayed. (**c**–**k**) Red and Orange fruit developmental parameters measured during stages of development (15 to 65 DAP). Line graphs display fruit weight (**c**–**e**), length to diameter ratio (L/D; **f**–**g**), length (**h**), and pericarp thickness (**i**–**k**). Mean values and standard error bars (*n* = 12 fruits from two experimental bays) are displayed. Bar plots depict the average of fruit weight (**e**), length (**h**), and thickness of pericarp (**k**) of Red and Orange fruits at the mature stage of development (65 DAP). Horizontal lines and stars denote statistical significance analyzed by one-way ANOVA (*: *p* ≤ 0.05, **: *p* ≤ 0.01, and ***: *p* ≤ 0.001). Abbreviations: Smart Glass Film (SGF); days after pollination (DAP).

The SGF did not impact fruit development in either cultivar (Figure 1a,b). Fruits of both the cultivars appeared green until 45 days after pollination (DAP), and matured to ripening stage 65 DAP, developing their respective red and orange colours. During fruit development, fruit weight, length to diameter ratio (L/D), and pericarp thickness were not significantly affected by SGF (Figure 1c–j). For both cultivars, the fruit weight reached its maximum at 45 DAP (Figure 1c–d), while the pericarp thickness continued to increase 45 DAP and peaked at 65 DAP (Figure 1h–i). The length to diameter ratio (L/D) increased from 15 DAP to 65 DAP, and the SGF slightly reduced the L/D ratio only in Red fruits at 35 and 55 DAP (Figure 1f–g). The two capsicum cultivars showed significantly different fruit morphological and anatomical traits (Table 1). Red fruits were heavier, shorter in length, and had a thicker pericarp compared to Orange fruits (Figure 1e,h,k). Overall, SGF did not affect morphology of mature harvestable fruits at 65 DAP.

**Figure 2:**
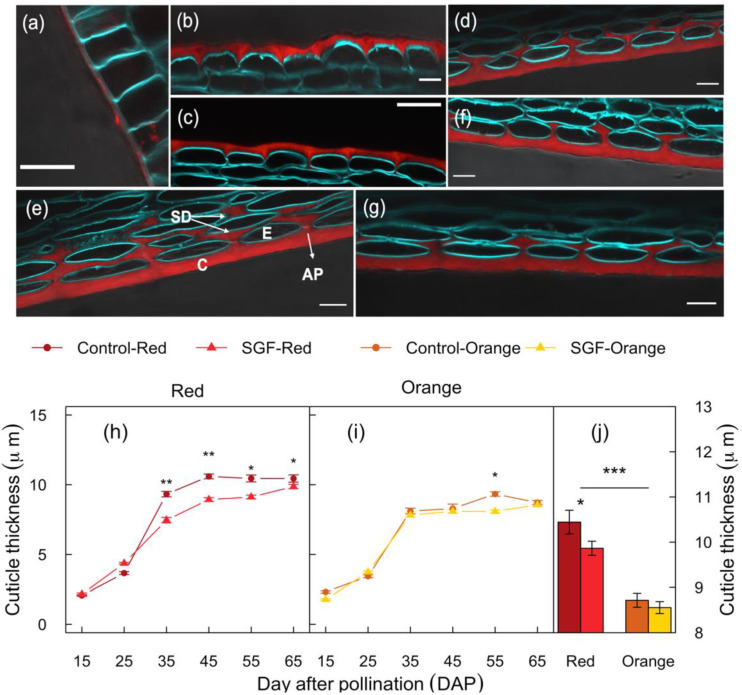
SGF impacts cuticle deposition during fruit development in a cultivar specific manner. (**a**–**g**) Confocal image panels showing the cuticle layer from the Red cultivar at 7 (**a**), 15 (**b**), 25 (**c**), 35 (**d**), 45 (**e**), 55 (**f**), and 65 (**g**) DAP. The lipophilic fluorescent dye Nile Red was used to stain the cuticle layer (red) and Calcofluor White M2R dye was used to stain cellulose in the cell walls (blue). Scale bar = 20 μm. (**h**–**i**) Cuticle thickness of fruits was quantified at different developmental stages using ImageJ. (**j**) The bar graph depicts the average cuticle thickness at the mature ripe stage (65 DAP) of Red and Orange cultivar development. Standard error bars are shown (*n* = 12). Horizontal lines and stars denote statistical significance analyzed by one-way ANOVA (*: *p* ≤ 0.05, **: *p* ≤ 0.01, and ***: *p* ≤ 0.001). Abbreviations: Cuticle (C), epidermal cell (E), anticlinal peg (AP), sub-epidermal deposit (SD), Smart Glass Film (SGF), days after pollination (DAP).

During early fruit development (seven DAP), a thin cuticle was formed in the fruit exocarp layer that extended to the anticlinal peg (AP) at 15 DAP (Figure 2a–b). Thereafter, cuticle deposition occurred around epidermal cells (25 DAP), leading to a rapid increase in thickness, while sub-epidermal deposition (SD) continued in the anticlinal plane from 45–65 DAP (Figure 2c–g). Cuticular deposition increased with fruit development, reaching a maximum thickness by 35–45 DAP (Figure 2h–i). Red fruits showed a 17.3% thicker cuticle compared to Orange fruits (Figure 2j; Table 1). The SGF significantly reduced cuticular thickness in Red fruits from 35 to 65 DAP (>5.6%), while in Orange fruits a 13.4% reduction was only observed at 55 DAP (Figure 2h–j). Therefore, the SGF caused a modest reduction in cuticle thickness of mature harvestable Red fruits (65 DAP), which had a much thicker cuticle compared to Orange fruits.

### 2.2. SGF Significatly Reduced Ascorbic Acid Content and Caused Subtle Effects on Fruit Quality Indexes

**Figure 3:**
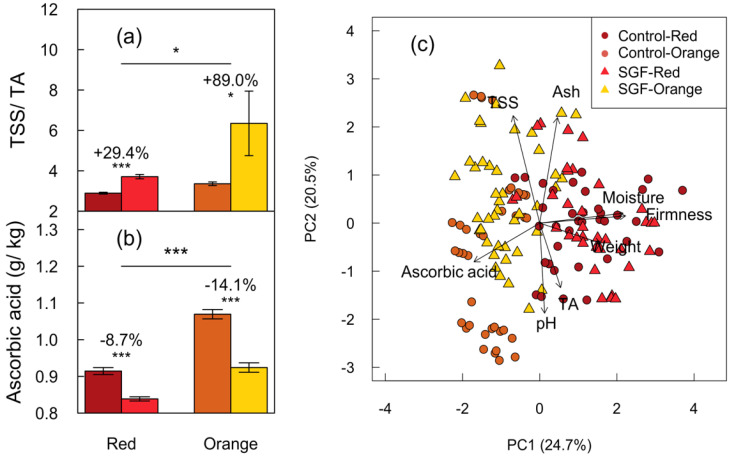
Analysis of postharvest quality parameters in Red and Orange fruits grown under SGF. (**a**) Total soluble solids (TSS) to titratable acids (TA) ratio (TSS/TA). (**b**) Ascorbic acid content in fruits (g/kg of fruit tissue). (**c**) Principal component analysis (PCA) showing multivariate associations among quality indexes. PC1 (24.7%) and PC2 (20.5%) define the cultivar specific differences in quality parameters. Standard error bars are shown (*n* = 12). Horizontal lines and stars denote statistical significance analyzed by one-way ANOVA (*: *p* ≤ 0.05 and ***: *p* ≤ 0.001).

SGF decreased ascorbic acid levels by 8.7% and 14% in both Red and Orange mature fruits (65 DAP), respectively (Figure 3b). SGF increased TSS in orange fruits by 4.4% and yet decreased titratable acidity by 21% in Red fruits (Table 1). Consequently, SGF significantly increased the TSS/TA ratio in Orange (+89%) more than Red (+29%) fruits (Figure 3a; Table 1). There was a significant decrease in moisture content (0.3%), increase in ash (6.5%), and reduction in colour parameters (a* 12%, b* 13%, and C* 12.5%) specific to Orange fruits (Table 1). SGF marginally increased pH in Red fruits. The SGF treatment did not affect total carotenoid content and fruit firmness in either cultivar. Therefore, SGF significantly reduced ascorbic acid levels in both varieties and had subtle effects on other fruit quality parameters in a cultivar-dependent manner.

Principal component analysis (PCA) of quality indexes revealed cultivar-specific differences in moisture, pH, ash, TA, ascorbic acid, and firmness (Figure 3c). Two primary axes of principal component (PC) variation cumulatively explained 45.2% of the variation. PC1 explained 24.7% of total variation that was positively associated with traits related to moisture, firmness, and fruit weight, and yet negatively associated with ascorbic acid. PC2 explained 20.5% of total variation associating with TSS, ash, pH, and titratable acidity. The Red cultivar was positively related to moisture, weight, and firmness, while the Orange cultivar was correlated with ascorbic acid. Fruit moisture significantly correlated with firmness and fruit weight. Overall, there is a greater separation of quality index traits by cultivar rather than SGF.

The relationship between altered photosynthetically active radiation (PAR; from 45 DAP to 65 DAP) and fruit quality traits were assessed using the Pearson’s correlation coefficient (Table 2). Red fruits showed a significant negative linear correlation relationship between PAR and colour parameters that was not observed for Orange fruits (Table 2). The SGF-related decrease in ascorbic acid in Orange fruits was also significantly correlated to PAR. Overall, the altered light transmittance by SGF led to significant correlation relationships between PAR and fruit colour (Red) or between PAR and ascorbic acid (Orange) in a cultivar-dependent manner.

### 2.3. SGF Did Not Impact the Shelf Life or Fruit Quality

**Figure 4:**
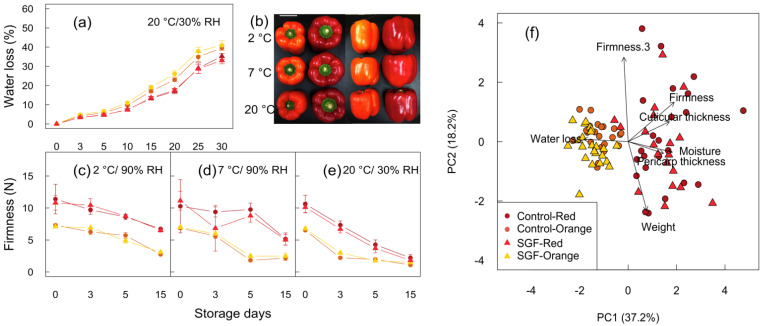
Assessment of shelf life by measuring water loss and firmness in mature ripe Red and Orange fruits from plants grown under SGF. (**a**) Line graph displays percentage water loss in fruits stored at 20 °C and 30% relative humidity (RH). (**b**) Appearance of fruits after 15 days of storage at different storage temperatures. (**c**–**e**) Line graph displays firmness (Newtons, N) of fruits stored at 2 °C and 90% RH (**c**), 7 °C and 90% RH (**d**), and 20 °C and 30% RH (**e**) after 0, 3, 5, and 15 days of storage. Scale bar = 5 cm. (**f**) Principal component analysis (PCA) showing multivariate associations among Firmness.3 (Firmness loss in day 3 stored 20 °C/30%RH), percentage water loss (day 3), cuticle and pericarp thickness parameters in both cultivars grown under SGF relative to the control. Standard error bars are displayed (*n* = 6–12). A one-way ANOVA did not reveal any significant differences (**a**,**c**–**e**).

SGF did not significantly impact the fruit water loss in either cultivar when fruits were stored at 20 °C and 30% relative humidity (RH, Figure 4a). Within the first three days of storage, the relative water loss from Red fruits (1.1%) was lower compared to Orange fruits (4.8%), which began to display signs of shriveling. Over 30 days, the total water loss was greater in Orange (−40%) compared to Red (−34%) fruits. There were differences in firmness between the two cultivars that were unaffected by SGF (Figure 4c–e). During 15 days of storage at 20 °C (30% RH), the firmness of Red and Orange fruits decreased by 81% (from 10.4 N to 2.0 N) and 82% (from 6.6 N to 1.2 N), respectively (Figure 4e). The industrial postharvest management practice to store capsicum fruits at 7 °C (90% RH) enhanced firmness of Red fruits, while decreasing temperature to 2 °C (90% RH) enhanced firmness of both fruit cultivars without signs of chilling injury (Figure 4b). Hence, an extended postharvest cooler temperature combined with higher relative humidity can enhance the firmness of Red and Orange fruits. Overall, the differences in shelf-life traits defined by fruit water loss and firmness were cultivar-dependent and not altered by SGF.

The principal component analysis of shelf-life parameters revealed links between the cultivar and impact of SGF. PC1 explained 37.2% of total variation (Figure 4f), which was positively associated with traits related to initial water loss in the first three days of postharvest storage. PC2 explained 18.2% of total variation and was positively associated with the initial three days of loss in firmness, but was negatively related to fruit weight. Red fruits trended towards greater firmness, higher moisture content, and a thicker cuticle deposition. Orange fruits trended towards faster water loss and reduced firmness quicker with a thinner cuticle. Therefore, the shelf life of fruits was largly unaffected by SGF and was cultivar dependent.

## 3. Discussion

New glasshouse covering materials such as SGF alter the light quantity and quality within the growth environment, thereby potentially affecting crop physiology, growth, and yield [7,13,38]. However, it was unknown whether SGF could also affect the development, ripening, and postharvest quality traits of mature fruit from different pigmented cultivars. The current study demonstrates that the altered light in SGF did not impact the development and ripening of Red and Orange fruits cultivated during the winter season. The effects of SGF on fruit quality parameters (e.g., colour, cuticle thickness, moisture content, ash, TSS, TA, and pH) were largely cultivar specific, and subtle changes in colour, TSS/TA, and ascorbic acid were within the range of marketable acceptance. We showed that SGF does not affect fruit water loss, firmness, or postharvest shelf life, revealing that the economic value of the fresh capsicum produce is maintained during storage.

### 3.1. SGF Does Not Affect Fruit Development, Ripening, or Appearance

Fruit development and ripening are important for early yield, and these marketable features are usually genetically and environmentally controlled [20,38]. During early stages of fruit development, additional red and far-red light promotes tomato fruit growth [20,39], and a reduction of PAR by covering materials can negatively affect the commercial fruit size of tomato fruits [21,22]. However, the decrease in PAR and previously reported decrease in red and far-red light caused by SGF [13], did not affect Red or Orange fruit morphology or ripening in this study. Rather, we found differences in fruit size, weight, and pericarp thickness during fruit maturation that were cultivar dependent. The thicker pericarp of Red fruits was associated with increased weight, which was consistent with a previous study [40]. There were subtle fluctuations in developmental parameters (e.g., weight, L/D) of fruits grown under SGF, which were not consistently observed during the 65 days of fruit development. Small differences in marketable fruit size may appear across a season [41], which could explain the subtle fluctuations in fruit development on plants adapting to the altered light transmission caused by SGF.

Carotenoid pigments provide Red and Orange capsicum fruits with their visual appeal and reflect the maturity of ripened fruits. Since capsicum fruit are non-climacteric and picked ripe, fruit colour does not usually change after harvest [42]. Carotenoid biosynthesis can be regulated by the light environment leading to changes in fruit colour [28]. It has been shown that carotenoid metabolism in orange and red varieties was responsive to changes in red, blue, and UV light spectrums [29,43,44]. While SGF did not affect the total carotenoid content in either variety, some colour parameters (e.g., L*, a*, b*, C* and *h*) were slightly lower in Orange fruits. This could be due to changes in individual carotenoid pigments that contribute to the colour variations in capsicum fruit cultivars [29,44]. Therefore, SGF does not appear to affect the marketable appearance of fruits from Red and Orange cultivars.

### 3.2. SGF Reduced Cuticular Deposition in the Red Cultivar

Cuticle deposition of the tomato fruit epidermis helps to protect inner mesocarp tissues from water loss [45,46]. Exposure of tomato fruits to different light conditions can also lead to variations in cuticle thickness [45,46]. We found that the process of cuticle development in capsicum was similar to tomato [46], in that during the early stage of capsicum development the cuticle is thinner and becomes thicker during ripening with an increase in sub-epidermal deposition. Cuticle thickness increased in Red and Orange capsicum fruits in a similar manner throughout development, yet SGF reduced cuticle deposition in Red fruits, indicating that light affects cuticle development in a cultivar-specific manner. UV radiation was positively correlated with cuticle deposition in tomato and sweet cherry fruits [46,47]. SGF was previously shown to reduce UV light transmission to an eggplant crop grown over both summer and winter seasons [13], and this could be the cause of the pronounced decrease in cuticular thickness 35 DAP in Red fruits. The decreased cuticle thickness in Red fruits grown under SGF did not impact water loss and hence postharvest self-life quality. Cuticle thickness is not a conventionally detectable parameter defining the marketability of fresh industrial capsicum produce [48], but may provide insight into shelf life.

### 3.3. SGF Reduced Ascorbic Acid Content

Lower ascorbic acid levels have been reported in tomato fruits and other fresh produce in growth environments with low UV light or that are shaded [49,50,51]. The blockage of 85% UV-B and/or 19% PAR by SGF [13] caused a notable decrease in ascorbic acid content in both Red and Orange capsicum fruits. There was a correlation between PAR and ascorbic acid content in Orange capsicum fruits, and similar cultivar specific effects have been reported for other fruit types [50,52,53]. Indeed, we found that fruit quality index traits were dependent on cultivar, rather than SGF. The accumulation of ascorbic acid provides a measure of the state of ripening as it can be correlated with the accumulation of cell wall polysaccharides and softening of the fruit pericarp [35,54]. However, SGF did not alter capsicum fruit ripening, despite the subtle reduction in ascorbic acid. The Red and Orange capsicum fruit cultivars grown under SGF provide an acceptable level of ascorbic acid (40~45 mg) that can be consumed as a recommended dietary allowance [55].

TSS can vary in fruits grown under different covering materials [7]. Altered far-red and infrared light transmission can affect sugar content differently depending upon the fruit crop. For example, a higher sugar content was observed in melon fruits grown under near infrared ray reducing nets [56], while additional far-red light improved sugar content in tomato fruits [39]. Reductions in PAR caused by flexible photovoltaic rooftop panels in tomato [21] and by SGF in eggplant [13] reduced TSS. We detected subtle changes in TSS and TA that were cultivar dependent. Overall, it was not surprising that the TSS/TA ratio was slightly higher in both Red and Orange capsicum fruit varieties given that SGF lowered ascorbic acid levels. The TSS/TA ratio reflects a taste sensory parameter used to determine fresh produce quality, but the slightly higher TS/TA ratio of capsicum fruits grown under SGF may not be sufficient to affect taste sensory preferences.

### 3.4. Fruit Firmness, Water Loss, and Shelf Life Storage Were Not Affected by SGF

The cuticle limits water loss through the fruit pericarp and capsicum fruits do not contain stomata on the epidermal surface [40,46]. We demonstrated that Red capsicum fruits have a thicker cuticle compared to the Orange variety, as previously reported [30,57], and further showed that this helps to retain water and extend fruit shelf life. While SGF reduced the cuticular thickness in Red fruits, the water loss during storage declined in a manner similar to fruits produced under the control glass. Capsicum fruits have a waxy surface that helps to prevent excess water loss [31,57]. There was a good correlation between fruit firmness, cuticle thickness, and higher moisture content in both fruit types. These traits determine how growers and distributors manage the postharvest storage environment to ensure high fresh produce quality for retail [30]. A higher relative humidity and lower temperature is commonly used to enhance the storage of green bell pepper fruits [58]. SGF did not impact fruit shelf life, nor the firmness and quality of Red and Orange fruits during long-term storage. We conclude that SGF does not have any major effects on the postharvest quality of fruits from Red and Orange capsicum cultivars.

## 4. Materials and Methods

### 4.1. Plant Materials, Growth Conditions, and Fruit Collection

The experiment was conducted in a controlled environment greenhouse facility with east–west orientation located on the Hawkesbury Campus of Western Sydney University, Richmond, NSW, Australia. The four research bays (105 m^2^ each) were fitted with HD1AR diffuse glass (70% haze, roof; 5% haze, wall), as previously described [13,59]. Two bays with diffuse glass were designated control bays and two bays (roof and side walls) were coated with SGF (ULR-80, Solar Gard, Saint-Gobain Performance Plastics, Sydney, Australia) and designated as treatment bays.

The experimental design involved a 2 × 2 factorial design testing two capsicum cultivars, Red and Orange (Gina and O06614, respectively, Syngenta, Durham, NC, USA). Seedlings were germinated for 42 days after sowing in Rockwool cubes (100 cm × 15 cm × 10 cm, Grodan, Roermond, The Netherlands) and transplanted (19th April and harvested 19th December 2019) into anchored Rockwool slabs contained within hydroponic gutters. Of the six gutters, the four middle gutters (10.8 m length × 0.25 m width, AIS Greenworks, Castle Hill, NSW, Australia) were alternatively planted with Red and Orange capsicum cultivars. The outer two gutters were planted with capsicum varieties, but not used for experimental data collection. Each slab supported four plants, such that each bay contained 40 experimental plant replicates. A trellis system was used to attain vertical plant growth. Plants were pruned to two main branches from the main stem that was allowed to grow 50 cm in height; weekly pruning was performed according to industry standards. Capsicum flowers were tagged upon evidence of pollination (August 2019) and fruits were harvested at 10-day intervals, starting 15 days after pollination (DAP) and ending at 65 DAP. A total of 12 samples from each cultivar per treatment at each fruit development stage were harvested, and the weight, diameter, length, and pericarp thickness were quantified.

Five PAR sensors (LI-190SZ Quantum Sensor, LI-COR) continuously logged environmental data. Environmental factors were monitored and controlled by the Priva system (Priva, The Netherlands) during the crop production process. Relative humidity (RH, 70/80%, day/night), temperature (25/20 °C, day/night), CO_2_ concentration (480–500 ppm), and nutrient solutions (EC: 2.5–3.0 dS/m, pH: 5.0–5.5) were maintained at optimal growth conditions as previously detailed [13].

### 4.2. Confocal Microscopy of Fruit Cuticle Thickness

Representative fruit samples were used to assess cuticle thickness using confocal microscopy, as previously described, with minor modifications [60]. Twelve different fruits from each genotype per treatment were harvested every 10 days. The exocarp cuticle from eight cross-sections of the equatorial region were quantified using an inverted laser scanning confocal microscope with a ×63 water immersion objective (Leica TCS SP5, Bensheim, Germany). Image processing was accomplished using LAS-AF 1.6.3 software. To avoid auto-fluorescence (cuticle, emission maximum = 475 nm), Nile red (Sigma Aldrich, St. Louis, MO, USA, 100 μg/mL methanol solution) and a cell wall-specific dye (Fluorescent bright 28; Calcofluor White M2R, Sigma Aldrich, St. Louis, MO, USA) dissolved in 0.01 M of distilled water were used to stain the samples for 10 min and 3 min, respectively. Excitation was set at 405 nm on the laser, and emission was collected between 420 and 436 nm (emission maximum at 428 nm) for the stained cell wall and between 597 and 649 nm (emission maximum at 620 nm) for the dyed cuticle. Two-dimensional and Z-stack images were obtained using a scan rate of 400 Hz in sequential scan mode to avoid interference between the two fluorophores. The step size was in the range of 1.0–1.5 μm and was determined via program auto-optimization, based on the input parameters of an individual Z-scan of the LAS-AF software (Leica Microsystems, Bensheim, Germany). The central region between pegs of the cuticle covering epidermal cells was used to estimate cuticle thickness and fruits were not affected by cuticle invaginations [32]. Image J (NIH, Bethesda, MD, USA) was used to determine the thickness of the cuticle (twelve biological replicates and eight technical replicates for each cultivar per treatment).

### 4.3. Postharvest Quality Trait Measurements

Fruit weighing at least 200 g were sampled 183 days after treatment (19 October 2019) and moisture content was determined [61]. Approximately 4 g of capsicum fruit equatorial pericarp was cut and oven dried at 70 °C for 48 h. Samples were cooled in a desiccator and weighed. This operation was repeated three times for each sample and expressed as a percentage. The pH of a liquid extract from 10 g of fruit sample (equatorial region) ground in 90 mL distilled water was determined [62]. Total soluble solids (TSS) were measured (three technical replicates per fruit and six biological replicates) using a digital refractometer (Mettler Toledo, Melbourne, Australia) using the liquid extract from the equatorial pericarp as previously described [62]. Ash was quantified using 3 g of the fruit equatorial zone and heated at 550 °C overnight into a grey-white ash as previously described [61]. Fruit colour was assessed upon harvest using a CR-400 chromameter (Minolta, Tokyo, Japan) calibrated against a standard white tile (Y = 84.8; x = 0.3199; y = 0.3377) prior to measurement [63]. Fruit surface colour measurements were taken at three points on the opposite sides to the equatorial region of the fruit. The Commission Internationale de l’Elcairage (CIE) L*, a*, b* colour scale was adopted, and the raw data was used to calculate chroma (C*) and hue angle (*h*) according to the formulas [C* = (a*^2^ + b*^2^)^0.5^] and [*h* = arctan (b*/a*)] as described [64]. Ascorbic acid was quantified (g of ascorbic acid per kg fresh sample) by titration using 2, 6-dichloro-indophenol as previously described with minor modification [52]. Ten grams of samples was blended, and the pulp was homogenized with 50 mL of 12% oxalic acid. Titratable acidity (TA) was determined by titration using NaOH (0.1 N) as a standardized solution according to [62]. The juice from 10 g of fruit sample was added to 90 mL of distilled water and homogenized in a blender. Each replicate liquid was titrated three times to determine an average of titratable acidity (TA, g of citric acid mg/g of fresh sample).

### 4.4. Carotenoid Quantification

65 DAP fruit samples (12 replicates) were harvested for both control and SGF treatments, snap-frozen using liquid N_2_, and stored at −80 °C. Carotenoids were extracted under low-light conditions with 500 μL extraction buffer (60% ethyl acetate: 40% acetone and 0.1% butylated hydroxytoluene (BHT)) as previously described [65]. The liquid was partitioned into the ethyl acetate layer by adding 500 μL of H_2_O, centrifuged to separate the carotenoid-containing organic phase, and saponified to liberate free carotenoid molecules. Saponified carotenoids were analyzed by reverse-phase high performance liquid chromatography (HPLC, Agilent 1200 Series) using the GraceSmart-C30 (4 μm, 4.6 × 250 mm column; Alltech) column, and absolute total carotenoid levels (mg/kg of fresh weight) were calculated as previously described [66,67].

### 4.5. Quantification of Fruit Firmness, Water Loss, and Shelf Life

The harvested fruit firmness was determined using the XT *plus* texture analyzer (Stable Micro Systems, Godalming, UK) as described with minor modification using the Probe Volodkevich Bite Jaws (HDP/VB) [64]. A 3 cm diameter hole in the pericarp was punched in the equatorial region of the capsicum by a stainless steel punch [64]. The fruit skin was placed down on the steel plate and parameters established (pre-test speed 2 mm/s, test speed 3 mm/s, post-test 2 mm/s, compression deformation 30%, and trigger force 0.05 N). Water loss was determined using twelve ripened fruits (65 DAP) from each cultivar and treatment, and stored in 20 °C, 30% RH for 30 days. A high vacuum grease (silicone lubricant, Dow Corning) was applied to the surface of fruits to cover the pedicel scars. Water loss was calculated as the percentage of weight loss based on the initial fruit weight at 3, 5, 10, 15, 20, 25, and 30 days post-storage [68]. Capsicum shelf life was assessed by counting the days until visual shriveling and fruit firmness was evident as previously described [40]. A total of 36 fruits were harvested from each cultivar and treatment were stored in 2 °C/90% (temperature/relative humidity), 7 °C/90% and 20 °C/30% for 30 days. During storage, six fruits were photographed, and firmness measured after 0, 3, 5, and 15 days.

### 4.6. Statistical Analysis

All data and statistical analyses were performed under R 4.0.0 statistical computing environment. A *p* ≤ 0.05 is considered statistical significance. Levene’s test from the *car* package analyzed the homogeneity of variance. Welch’s *t*-test for unequal variances (with ≤0.05 probability for Levene’s test) used the *oneway.test* function in R. The normal distribution was tested using the Shapiro–Wilk test for normal distribution, and statistically significant (≤0.05) parameters were then analysed using the Kruskal–Wallis test, which is a non-parametric equivalent of one-way ANOVA. Repeated measures ANOVA on different time point of measurements, such as fruit development, water loss, and firmness changes, used Mauchly’s test (*p* ≤ 0.05), and pairwise comparison by the *anova_test* function from the *rstatix* package. Other packages were also used, including (but not limited to) *Lubridate* (for effective use of dates in plots), *Sciplot* (for plotting), and *doBy* (for calculating means and standard errors). The Pearson’s correlation coefficient of PAR and quality parameters used the *rcorr* function of the *Hmisc* package. Principal component analysis (PCA) was used to test the multivariate associations among quality indexes, and the shelf-life-related parameters used the *prcomp* function.

## Figures and Tables

**Table 1:** Effects of SGF and cultivar interactions on morphology and postharvest quality traits from Red and Orange mature ripe fruits (65 DAP). Data represents the average from multiple fruits (*n* = 12) and the standard error of mean is displayed in brackets. Statistical analyses were performed using a one-way ANOVA (*: *p* ≤ 0.05, **: *p* ≤ 0.01, and ***: *p* ≤ 0.001). Abbreviations: Not significant (NS), smart glass film (SGF), percentage difference (%), cultivar (CV), days after pollination (DAP), total soluble solids (TSS), titratable acids (TA), chroma (C*), hue angle (*h*), lightness (L*), red/green value (a*), yellow/blue value (b*), fresh weight (FW).

Trait	Red Variety				Orange Variety				CV	SGF × CV
	Control	SGF	%	SGF	Control	SGF	%	SGF		
Morphological Parameters in Ripe Fruit (65 DAP)										
Weight (g)	237 (±37)	243 (±45)	+2.3	NS	213 (±21)	222 (±27)	+4.4	NS	*	NS
Diameter (mm)	77.6 (±9.0)	76.3 (±6.9)	−1.7	NS	78 (±8)	78 (±8)	−0.4	NS	NS	NS
Length (mm)	81.3 (±7.6)	80.6 (±5.1)	−0.8	NS	85.3 (±5.8)	86.5 (±3.8)	+1.4	NS	**	NS
Pericarp thickness (mm)	7.5 (±0.7)	7.5 (±0.6)	0	NS	6.9 (±0.4)	7.1 (±0.3)	+2.9	NS	**	NS
Cuticular thickness (µm)	10.4 (±0.3)	9.9 (±0.1)	−5.6	*	8.7 (±0.2)	8.6 (±0.1)	−1.1	NS	***	NS
Quality parameters in ripe fruit (65 DAP)										
Moisture content (%)	92.9 (±0.1)	93.1 (±0.1)	+0.2	NS	92.4 (±0.05)	92.1 (±0.1)	−0.3	**	***	**
pH	5.04 (±0.01)	5.08 (±0.01)	+0.8	***	5.06 (±0.01)	5.05 (±0.00)	−0.2	NS	NS	**
Ash (g/kg)	3.2 (±0.01)	3.2 (±0.01)	0	NS	3.1 (±0.01)	3.3 (±0.01)	+6.5	***	NS	**
Total soluble solids (TSS, %)	6.61 (±0.06)	6.62 (±0.11)	+0.2	NS	6.57 (±0.07)	6.86 (±0.12)	+4.4	*	NS	NS
Titratable acidity (TA, g/kg FW)	2.29 (±0.15)	1.82 (±0.26)	−21	*	2.00 (±0.15)	1.84 (±0.26)	−8	NS	*	**
TSS/TA	2.89 (±0.04)	3.71 (±0.11)	+29	***	3.35 (±0.09)	6.35 (±1.60)	+89	*	*	NS
L* (Lightness)	70.6 (±0.3)	70.6 (±0.2)	0	NS	75.3 (±0.35)	74.8 (±0.6)	−0.6	NS	***	NS
a* (-green to +red)	17.9 (±0.5)	17.4 (±1.0)	−2.5	NS	17.0 (±0.9)	14.9 (±0.2)	−12	**	**	*
b* (-blue to +yellow)	−1.1 (±0.3)	−1.4 (±0.4)	−27	NS	7.1 (±0.6)	6.2 (±0.7)	−13	*	***	NS
C*	17.9 (±0.3)	17.5 (±0.6)	−2.2	NS	18.4 (±0.4)	16.1 (±0.2)	−12.5	**	NS	*
*h*	−0.06 (±0.01)	−0.08 (±0.02)	−33.3	NS	0.40 (±0.01)	0.40 (±0.02)	0	NS	***	NS
Ascorbic acid (g/kg FW)	0.91 (±0.07)	0.83 (±0.04)	−9	***	1.07 (±0.08)	0.92 (±0.09)	−14	***	***	**
Firmness (N)	11.6 (±0.6)	10.5 (±0.4)	−9.4	NS	6.9 (±0.1)	7.0 (±0.1)	+0.9	NS	***	NS
Total Carotenoids (mg/kg FW)	482 (±65)	501 (±41)	+3.8	NS	458 (±45)	362 (±60)	−21	NS	NS	NS

**Table 2:** Pearson’s correlation coefficient analysis of photosynthetically active radiation (45 to 65 DAP) with fruit quality indexes in mature ripe fruit (65 DAP). The correlation coefficient and probability values were analyzed by R using “Hmisc” package. Data was analysed from multiple fruits (*n* = 12). Statistical analysis was performed using a one-way ANOVA (*: *p* ≤ 0.05 and **: *p* ≤ 0.01). Abbreviations: Smart glass film (SGF), days after pollination (DAP), total soluble solids (TSS), titratable acids (TA), photosynthetically active radiation (PAR), chroma (C*), hue angle (*h*), lightness (L*), red/green value (a*), and yellow/blue value (b*).

Correlation	Red		Orange	
	Control	SGF	Control	SGF
PAR-TSS	−0.22	0.47	0.11	−0.62
PAR-TA	0.10	−0.12	−0.69	0.37
PAR-Moisture	0.24	−0.25	0.14	0.26
PAR-pH	−0.11	−0.76	−0.56	0.33
PAR-Ascorbic acid	−0.61	−0.22	0.12	0.93 **
PAR-Ash	−0.58	−0.40	−0.18	0.08
PAR-L*	0.14	−0.97 **	0.35	0.52
PAR-a*	0.05	−0.91 *	−0.01	0.24
PAR-b*	0.01	−0.94 *	0.07	0.38
PAR-C*	0.05	−0.91 *	0.01	0.35
PAR-*h*	0.02	−0.92 *	0.11	0.38
PAR-Total Carotenoid	0.09	0.37	0.47	0.55

## Data Availability

Not applicable.
